# The maternal and early embryonic transcriptome of the milkweed bug *Oncopeltus fasciatus*

**DOI:** 10.1186/1471-2164-12-61

**Published:** 2011-01-25

**Authors:** Ben Ewen-Campen, Nathan Shaner, Kristen A Panfilio, Yuichiro Suzuki, Siegfried Roth, Cassandra G Extavour

**Affiliations:** 1 Department of Organismic and Evolutionary Biology, Harvard University, 16 Divinity Avenue, Cambridge, MA 02138, USA; 2 Monterey Bay Aquarium Research Institute, 7700 Sandholdt Road, Moss Landing, CA 95039, USA; 3 Institute for Developmental Biology, University of Cologne, Cologne Biocenter, Zülpicher Straße 47b, 50674, Cologne, Germany; 4 Department of Biological Sciences, Wellesley College, 106 Central Street, Wellesley MA 02481, USA

## Abstract

**Background:**

Most evolutionary developmental biology ("evo-devo") studies of emerging model organisms focus on small numbers of candidate genes cloned individually using degenerate PCR. However, newly available sequencing technologies such as 454 pyrosequencing have recently begun to allow for massive gene discovery in animals without sequenced genomes. Within insects, although large volumes of sequence data are available for holometabolous insects, developmental studies of basally branching hemimetabolous insects typically suffer from low rates of gene discovery.

**Results:**

We used 454 pyrosequencing to sequence over 500 million bases of cDNA from the ovaries and embryos of the milkweed bug *Oncopeltus fasciatus*, which lacks a sequenced genome. This indirectly developing insect occupies an important phylogenetic position, branching basal to Diptera (including fruit flies) and Hymenoptera (including honeybees), and is an experimentally tractable model for short-germ development. 2,087,410 reads from both normalized and non-normalized cDNA assembled into 21,097 sequences (isotigs) and 112,531 singletons. The assembled sequences fell into 16,617 unique gene models, and included predictions of splicing isoforms, which we examined experimentally. Discovery of new genes plateaued after assembly of ~1.5 million reads, suggesting that we have sequenced nearly all transcripts present in the cDNA sampled. Many transcripts have been assembled at close to full length, and there is a net gain of sequence data for over half of the pre-existing *O. fasciatus *accessions for developmental genes in GenBank. We identified 10,775 unique genes, including members of all major conserved metazoan signaling pathways and genes involved in several major categories of early developmental processes. We also specifically address the effects of cDNA normalization on gene discovery in *de novo *transcriptome analyses.

**Conclusions:**

Our sequencing, assembly and annotation framework provide a simple and effective way to achieve high-throughput gene discovery for organisms lacking a sequenced genome. These data will have applications to the study of the evolution of arthropod genes and genetic pathways, and to the wider evolution, development and genomics communities working with emerging model organisms.

[The sequence data from this study have been submitted to GenBank under study accession number SRP002610 (http://www.ncbi.nlm.nih.gov/sra?term=SRP002610). Custom scripts generated are available at http://www.extavourlab.com/protocols/index.html. Seven Additional files are available.]

## Background

New and emerging model organisms occupy an increasingly important part of the developmental biology and developmental genetics research landscape. While studying a huge diversity of animals has long been the norm in the classical fields of experimental embryology and functional morphology [see for example [1-3]], the molecular biology revolution and the advent of the "model system" concept [4] created demand for a small number of highly genetically manipulable organisms that could be intensively studied [5]. Research on these "big six" [sensu 6] genetic model organisms has led to enormous advances in our understanding of general principles of embryogenesis. However, placing these general principles in an evolutionary context requires broader taxonomic sampling. Many researchers have highlighted the need for developing new model organisms for specific comparative, evolutionary and ecological questions [6-8]. It has also been suggested, however, that the single gene expression approach of the last several decades of evolutionary developmental biology ("evo-devo") has outlived its usefulness, and that what are needed are not more model organisms, but rather a smaller number of groups chosen for the ability to functionally manipulate genes [9,10]. Sophisticated gene expression techniques and even stable germline transgenesis have been developed in a large array of models outside of the "big six" [see for example [11,12]]. The ancient history of the small RNA processing machinery [13,14] means that gene knockdown is a feasible goal for most organisms, as long as the sequences of genes of interest are available.

While whole genome sequencing is an increasingly viable option for some organisms, many new models, particularly within the arthropods, lack the large community resources necessary to finance and maintain annotation of a genome. For these reasons, many researchers studying non-traditional model organisms have turned to Sanger-sequenced EST libraries [see for example [15,16]]. In principle this method of gene discovery can lead to high-throughput expression and functional genetic analyses of multiple genes [see for example [17]]. In practice, however, most non-traditional organism studies are still subject to a gene discovery bottleneck. This is largely because at the scale needed to uncover rare developmental transcripts, Sanger-based EST sequencing quickly becomes technically and financially prohibitive for many labs working on organisms with smaller research communities. In addition, those smaller-scale EST projects that have been carried out are often not publically available in easily searchable formats, and their potential contribution to the developmental and evolutionary biology fields is thus limited.

Next-generation sequencing (NGS) offers comparative and evolutionary developmental biologists a way to obtain orders of magnitude more developmental gene data than ever before, at a fraction of its former cost. Several studies have demonstrated the feasibility of NGS for identifying SNPs for population studies and gene sequences for use as phylogenetic markers [18-35]. Unfortunately, the lack of suitable protocols for cDNA preparation, and of established pipelines for analysis have left this tool under-utilized by many evo-devo researchers. Furthermore, according to some estimates [35], few of these studies have been carried out at a scale large enough to provide significant recovery of rare transcripts, and therefore of developmental genes. Here we present an optimized protocol for synthesizing cDNA for 454 Titanium pyrosequencing, as well as a simple workflow for *de novo *assembly of the data without a reference genome, annotation and analysis of the dataset, and a demonstration of its utility for comparative developmental genetics.

A large body of literature is dedicated to the development and genomics of holometabolous insects (insects undergoing complete metamorphosis between embryonic and adult stages). Tens of holometabolous insect genomes are now available, thanks largely to work on *Drosophila melanogaster*, other drosophilids, and dipteran disease vectors [36,37]. In contrast, relatively little is known about the development of hemimetabolous insects, which undergo incomplete metamorphosis. Although several of these insects are amenable to laboratory culture and a variety of experimental manipulations, molecular developmental studies are scarce, and gene discovery rates remain low. Notable exceptions among the Hemiptera are the aphid *Acyrthosiphon pisum *and the Chagas' disease vector *Rhodnius prolixus*, whose genomes are completed and in progress respectively [38,39]. However, the aphid genome has undergone extensive duplications and gene loss, possibly due to its unusual reproductive and ecological characteristics [38]. The mammalian blood feeding needs of *R. prolixus *make it a sub-optimal organism for developmental studies.

**Figure 1:**
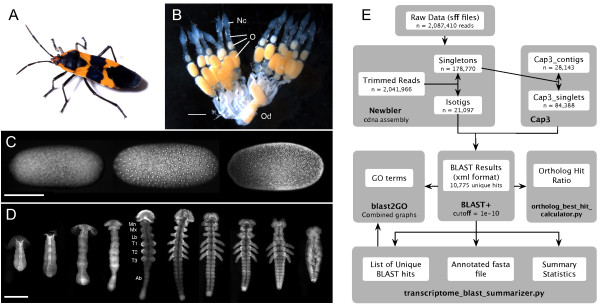
**Introduction to *Oncopeltus fasciatus *and the workflow for producing a *de novo *transcriptome assembly**. (*A*) An adult milkweed bug, *Oncopeltus fasciatus*. (*B*) Ovaries of adult female. Anterior is up. Oocytes (O) are visible in progressive stages of growth before reaching a common oviduct (Od). Oocytes are cytoplasmically connected to nurse cells (Nc) in the anterior of each ovariole. Scale bar = 1.0 mm. (*C*-*D*) The stages of *O. fasciatus *embryogenesis represented in this transcriptome. Embryos are stained with Sytox Green (Invitrogen) to visualize nuclei. Scale bars = 0.5 mm. (*C*) Development proceeds from left to right. Anterior is to the left. The cellularized blastoderm forms during the first ~20% of development (~0-24 hours at 28°C), as nuclei reach the surface of the yolk and repeatedly divide. *(D*) Germ band extension and segmentation occur from ~20-60% of development (~24-72 hours at 28°C). Development proceeds from left to right. Anterior is up. Mn = mandibular segment; Mx = maxillary segment; Lb = labial segment; T1-T3 = leg-bearing thoracic segments 1-3; Ab = abdomen. (*E*) The flow of information during this *de novo *transcriptome assembly project. Data files are represented as white boxes within grey boxes that indicate the computer programs used to generate these files. All of the computer programs used are freely available. Ortholog_best_hit_calculator.py and transcriptome_blast_summarizer.py are custom python scripts available at http://www.extavourlab.com/protocols/index.html (see text for details). Photograph in (*A*) courtesy of David Behl.

The milkweed bug *Oncopeltus fasciatus *(Figure 1A-D) has emerged as a promising hemipteran system for studying the molecular development of hemimetabolous insects [40-42]. It can be reared easily and cheaply in the laboratory, and has a long history as a laboratory animal for classical embryology and pattern formation studies [43-45]. More recently, robust protocols for *in situ *hybridization, live imaging of embryogenesis, and RNAi-mediated gene knockdown have been developed and successfully applied to the study of the evolution of development [see for example [46,47]].

Here we present the results of the sequencing and *de novo *assembly of the *Oncopeltus *ovarian and early embryonic transcriptome. We outline an assembly and analysis framework using a combination of existing tools and freely available custom-made command line computational tools, which we hope will make this approach to gene discovery accessible to comparative developmental biologists. We identify homologues of genes involved in all major signaling pathways and developmental processes, including biologically verified splicing isoforms for some genes. We also address the need for library normalization in these studies, and show that at large enough scales of NGS, large numbers of developmental genes can be discovered even with omission of a normalization step.

## Results and Discussion

### *Assembling the ovarian and embryonic transcriptome of *O. fasciatus

**Figure 2:**
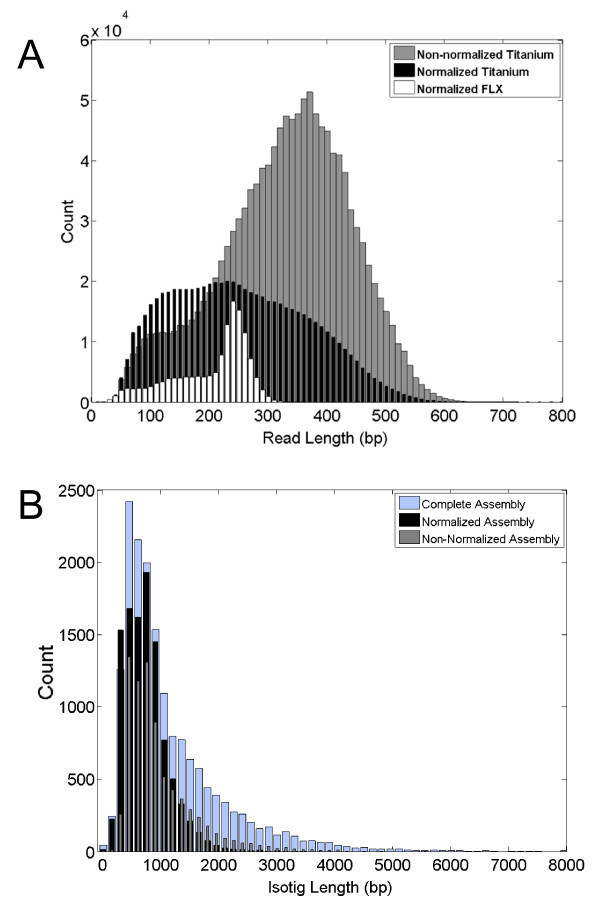
**Effects of normalization and 454 sequencing chemistry on read length and isotig length**. (*A*) Titanium sequencing chemistry (grey, black) generally results in longer read lengths when compared with FLX chemistry (white). However, the normalized sample run with Titanium chemistry (black) had shorter read lengths than the non-normalized sample (grey). This result is likely due to a technical error in that particular sequencing run, since a 1/8 plate run of the same sample showed a read length distribution comparable to that of the non-normalized sample (Additional file 1). (*B*) Isotig length distributions from assemblies of Titanium-sequenced data. The longest isotig per isogroup is shown. The number of bases in the non-normalized (grey) and normalized (black) samples has been equalized to eliminate possible bias due to the greater number and length of reads obtained from the run of the normalized sample (see (*A*)). The isotigs generated from the normalized cDNA tended to be shorter than those produced by the non-normalized cDNA (see also Table 2). Pooling all FLX and Titanium reads generates an assembly with more, longer isotigs (blue).

We prepared cDNA from ovaries and early to mid-staged embryos of *O. fasciatus*, covering oogenesis and all major stages of embryonic patterning (Figure 1B-D). These cDNA samples were prepared using a protocol optimized for preparation of small or limiting samples for 454 pyrosequencing (see Materials and Methods). From these libraries, we generated a total of 2,087,410 sequence reads (Table 1). This includes reads generated using GS-FLX technology as well as both normalized (N) and non-normalized (NN) cDNA sequenced using the GS-FLX Titanium platform. As expected, the reads generated using GS-FLX Titanium technology were substantially longer than those generated using GS-FLX technology (Table 1, Figure 2A). However, the N sample gave an unexpectedly low number of reads, which were on average shorter than those generated by the NN sample (Table 1; Figure 2A). Given that a pilot run of one lane (1/8 plate) of this same normalized cDNA sample generated roughly equal number and size-distribution as a NN pilot study (Additional file 1), we suspect that a technical error reduced the sequencing efficiency of this plate. Despite the comparatively low yield of this normalized cDNA, it still generated more than 600,000 high quality reads that we therefore included in subsequent analyses.

**Table 1:** Sources of *O. fasciatus *sequence reads.

Tissue	Normalized?	cDNA prep	454 Platform	No. Plates	No. Reads	Median Read Length	Accession #
Ovary	Y	SMART	GS-FLX	¼	65,394	225	SRR057570.2
Embryonic	Y	SMART	GS-FLX	¼	71,911	230	SRR057571.1
Ovarian and Embryonic	Y	Modified SMART	GS-FLX Titanium	1 + ¼	656,782	244	SRR057572.1
Ovarian and Embryonic	N	Modified SMART	GS-FLX Titanium	1 + 1/8	1,293,323	313	SRR057573.1
**Total**				**2 + 7/8**	**2,087,410**	**301**	SRP002610.1

We used the cDNA assembly algorithm of Newbler v2.3 (Roche) to screen the reads for adaptor sequence and then assemble the cleaned reads (see Note Added in Proof for a comparison with Newbler v2.5). After quality trimming and adapter screening, 2,041,966 reads (97.8%) were used in the assembly. Of these, 1,773,450 (86.9%) assembled either wholly or partially into contigs, and 178,770 (8.8%) remained as singletons. The remaining reads were excluded as either originating from repeat regions (9,875 reads; 0.05%), outliers (26,943 reads; 1.3%), or too short (<50 base pairs: 52,928 reads; 2.6%).

To our knowledge, Newbler v2.3 and higher are the only assembly programs that address alternative splicing and can output multiple isoforms per gene. Newbler v2.3 explicitly accounts for alternative splicing by creating a hierarchical assembly composed of three elements: contigs, isotigs, and isogroups. For consistency, we follow their terminology. Contigs are stretches of assembled reads that are free of branching conflicts. In other words, contigs can be thought of as exons or sets of exons that are always co-transcribed. Isotigs represent a particular continuous path through a set of contigs, i.e. a transcript. An isogroup is the set of isotigs arising from the same set of contigs, i.e. a gene. Different isotigs within an isogroup are thought to represent alternative isoforms of the same gene. Note that it is possible for an isogroup to contain only one isotig, and it is also possible for an isotig to be composed of only one contig.

After the initial Newbler assembly, we noticed substantial redundancy among the singletons. We therefore subjected the 178,770 unassembled singletons to a secondary assembly with CAP3 [48]. This secondary assembly reduced the number of singletons from 178,770 to 112,531 (28,143 cap3_contigs and 84,388 cap3_singlets). Thus, in total, our assembly generated a total of 133,628 sequences, including isotigs, cap3_contigs and cap3_singlets (Table 2).

**Table 2:** *O. fasciatus *transcriptome assembly statistics.

	Full Assembly	Normalized Assembly	Non-Normalized Assembly
Assembled reads (base pairs)	1,773,450 (508,738,047)	389,605 (84,353,140)	336,568 (108,372,883)
Isogroups ("genes")	16,617	10,581	7,591
Isotigs ("transcripts")	21,097	11,353	8,346
Isotig N50	1,735	846	1,162
Mean # isotigs per isogroup	1.3	1.1	1.1
Contigs ("exons")	22,235	11,839	8,731
Mean # contigs per isotig	1.9	1.2	1.3
Singletons (singletons after secondary CAP3 assembly)	178,770 (112,531)	110,265 (N/A)	52,585 (N/A)

To enable comparison, we equalized individual assemblies of Normalized and Non-Normalized samples to contain the same number of base pairs before assembly.

Our data assembled into 22,235 contigs, organized among 21,097 isotigs (Figure 2B). The isotig N50 length was 1,735 bp (in other words, 50% of the bases are incorporated into isotigs ≥ 1,735 bp), and 14,460 (68.5%) of the isotigs contained only one contig. The 21,097 isotigs fell into 16,617 isogroups, of which 14,562 (87.6%) contain only one isotig (average number of isotigs per isogroup = 1.3).

The average coverage among contigs was 23.2 reads/bp (median coverage = 6.9 reads/bp) (Additional file 2). This coverage value is more than twice as high as the highest reported value from a *de novo *transcriptome assembly to date [summarized in [20]]. Such deep coverage should be helpful for overcoming the presence of insertion/deletion errors in the individual raw reads [49].

To test whether our assembly would have been aided by the inclusion of nucleotide sequence from *Rhodnius prolixus*, the most closely related hemipteran to *O. fasciatus *whose genome is currently being sequenced [39], we used the BLASTN algorithm to compare our isotigs (the longest isotig per isogroup) with the published ESTs of *R. prolixus *with an e-value cut-off of 1e-6. Consistent with previous observations of extremely low levels of conservation between insect genomes [50] we found that only 53 out of 16,617 isotigs had hits to *R. prolixus *ESTs on the nucleotide level. These results suggest that *de novo *sequencing and assembling efforts will be necessary for most insect species, even when sequence data are available for other members of the same order. We note, however, that a recent study [51] has shown that it may be possible to incorporate EST data from different species into a *de novo *assembly by using amino acid sequence rather than nucleotide sequence.

### Validation of predicted alternate isoforms

**Figure 3:**
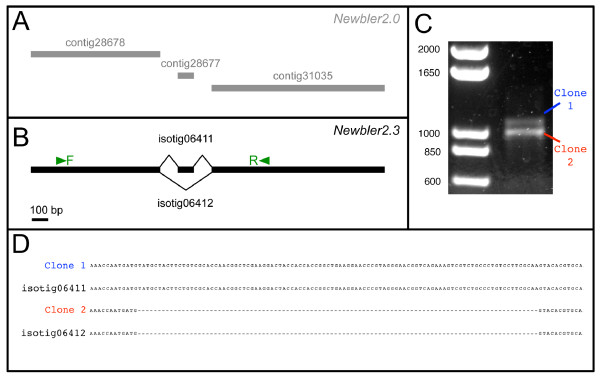
**Newbler 2.3 correctly identifies splicing isoforms of *nanos***. (*A*) Newbler v2.0 identified three separate contigs that map to an *O. fasciatus nanos *homologue that we had previously identified by degenerate PCR (Ewen-Campen & Extavour, unpublished). Newbler v2.0 failed to identify these contigs as belonging to the same transcript because of branching conflicts amongst the reads joining these contigs. BLASTX against the RefSeq protein database identified only contig 31035 as being a putative *nanos *homologue; the other two contigs lie outside the conserved Nanos domain and obtain no BLAST hits. (*B*) Newbler v2.3 predicted that the same three contigs identified by Newbler v2.0 belonged to two isotigs, or splicing isoforms. (*C*) RT-PCR with specific primers F and R shown in (*B*) resulted in two bands of the predicted sizes of the isotigs predicted by Newbler v2.3. (*D*) Sequencing the bands from (*C*) revealed that they were identical to the sequences of the predicted isotigs from (*B*).

To examine whether the alternative isoforms predicted by Newbler v2.3 are in fact present in developing embryos of *O. fasciatus*, we first focused on a gene of particular interest to developmental biologists, *nanos*. This conserved metazoan gene was first described as a loss of function mutation in *Drosophila melanogaster *[52], and is necessary for germ cell and posterior somatic development [reviewed in [53]]. Newbler v2.3 predicted this gene to encode two alternative isotigs within a single isogroup (Figure 3B). The two isotigs differ in that the longer contains an additional 100-bp exon that is absent from the shorter (Figure 3B). We designed PCR primers against sequences present in both isotigs (Figure 3B arrows), which amplified two bands differing by ~100 bp from a pool of embryonic cDNA (Figure 3C). Sequencing of these two bands confirmed that they differ exactly as predicted by Newbler v2.3 (Figure 3D).

Importantly, a previous version of Newbler (v2.0), which does not account for alternative splicing, failed to join together the three fragments which were linked by Newbler v2.3 (Figure 3A). Because of this, Newbler v2.0 (and presumably other assemblers which do not address branching within contigs) predicted three separate contigs, only one of which could be identified as *nanos *with BLASTX, as the others fall in poorly conserved regions of the gene. Thus, the ability of Newbler2.3 to handle branching conflicts between reads allows this program to assemble longer continuous sequences, which are therefore in turn more easily annotated using BLAST.

To further characterize the accuracy of Newbler's predictions of alternative transcript isoforms, we randomly selected 10 isogroups that contained exactly two alternative isotigs differing by the presence/absence of a single contig (Additional file 3). As we did for *nanos*, we designed primers to flank the region differing between the two predicted isoforms (Additional file 3A), and performed RT-PCR on *O. fasciatus *embryonic cDNA. In eight of ten instances, we observed bands of the predicted sizes following agarose gel electrophoresis (Additional file 3B,C). However, in four of the eight positive cases, additional, unpredicted bands were present (Additional file 3). In one of the ten cases, we observed two RT-PCR products, but only one of them was of the predicted size (Additional file 3C, lane 6). Taken together, these results suggest that Newbler v2.3 has a low rate of false positives in the prediction of multiple splicing isoforms. Including our investigation of *nanos*, only one of 11 test cases (9.1%) produced a single RT-PCR product where Newbler v2.3 had predicted multiple products. However, we observed that roughly half of the time, Newbler v2.3 failed to predict all of the isoforms identified via RT-PCR.

### Transcriptome annotation

A BLASTN search of our dataset for the 93 existing GenBank accessions for *O. fasciatus *sequences yielded a hit result for 56% of the accessions, with an e-value cut-off of 1e-10. This result may be due in part to the short length of some of the GenBank sequences. Accordingly, we found that accessions with hits in the database were significantly longer (mean length 729 bp) than accessions without hits (mean length 397 bp) (unpaired Student's *t*-Test: *t *= 2.89, DF = 91, *p *= 0.0048). Of greater relevance to developmental applications of this dataset, however, was our finding that 85% of *O. fasciatus *developmental genes with existing GenBank accessions (n = 32) are represented in our transcriptome.

We then used BLASTX to map the 133,628 *O. fasciatus *sequences (isotigs, cap3_contigs and cap3_singletons) against the entire RefSeq Protein database with an e-value cut-off of 1e-10. To simplify these statistics, we report only the BLAST results for the longest isotig per isogroup, under the assumption that all isotigs within an isogroup share nearly identical BLAST results. Of 16,617 isotigs, 7,219 (43.4%) had at least one hit. Of the 28,143 cap3_contigs, 2,594 (9.2%) had hits, and of the 84,388 cap3_singlets, 2,367 (2.8%) had hits. These values are higher than comparable BLAST statistics of most other published studies of 454-generated *de novo *transcriptomes [24-26,30,32,33], likely because deeper sequencing increases the length of assembled sequences and thereby makes these sequences more likely to be identified via BLAST. The unidentifiable sequences likely originate from UTRs or non-conserved portions of protein-coding sequences. Of the top BLAST hits, 89.3% were genes from arthropod sequences (Additional file 4). Of the 12,180 *O. fasciatus *sequences with BLAST hits, 1,455 hit non-overlapping segments of the same top BLAST hit (i.e. potentially unassembled portions of the same transcript), and 825 hit overlapping segments of the same top BLAST hit (i.e. potential paralogs). Excluding those 1,455 potentially double-counted BLAST hits, our transcriptome identified a total of 10,775 genes. The assembled sequences generated in this study, as well as pre-computed BLAST results, are available as flat files from the authors upon request.

**Figure 4:**
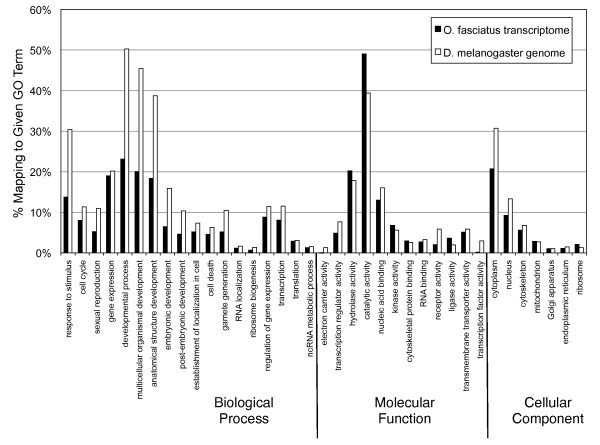
**GO term distribution of BLAST hits from the *O. fasciatus *transcriptome compared with those from the *D. melanogaster *genome**. Several GO categories are shown within the top-level divisions of Biological Process, Molecular Function, and Cellular Component. Column heights reflect the percentage of annotated sequences in each assembly that mapped to a given Biological Process GO term. The relative percentages of genes falling into GO categories are comparable between our *O. fasciatus *transcriptome (black) and the *D. melanogaster *transcriptome (white).

To explore and summarize the functional categories of the genes sequenced in this study, we obtained the Gene Ontology (GO) terms associated with the top 20 BLAST hits of each sequence using Blast2GO [54]. Among the 7,059 genes for which we obtained GO terms, we observed a wide diversity of functional categories represented on all levels of the Gene Ontology database (Figure 4). The *O. fasciatus *sequences fall into GO categories with a roughly similar distribution to that of the well-annotated *Drosophila melanogaster *genome, suggesting that our sequence data contain a large diversity of genes involved in a variety of biological processes, and do not contain any notable biases towards particular categories of genes.

### *Assessing coverage of the *O. fasciatus *transcriptome*

We wished to know how thoroughly our sequencing efforts sampled the true diversity of transcripts present in our cDNA samples. This is a two-part question: first, of the genes truly expressed during *O. fasciatus *oogenesis and embryogenesis, how many did we identify? And second, of these identified genes, how thoroughly had we assembled their full-length transcripts?

**Figure 5:**
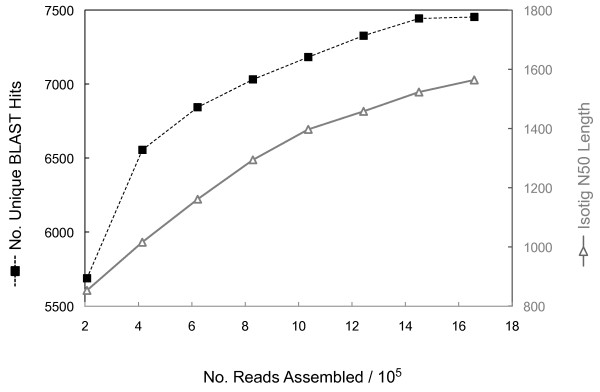
**Assessing coverage of the *O. fasciatus *transcriptome**. Randomly chosen subsets of increasing numbers of Titanium reads were used to generate progressively larger sub-assemblies. The number of reads in each sub-assembly (X axis) is plotted against the number of unique BLAST hits in each sub-assembly (left Y axis: black), and against the N50 isotig length (right Y axis: grey). For this analysis BLAST was performed against the SwissProt database. The number of unique BLAST hits plateaus when the assembly is composed of approximately 1.5 million reads. However, the N50 isotig length maintains an approximately constant rate of increase.

To address the first question, we created eight separate assemblies of progressively larger sub-samples of our total reads and tallied the total number of genes identified via BLASTX. The number of newly discovered genes began to plateau after ~1.5M reads (1 7/8 plates in our case) (Figure 5 black line). However, the N50 isotig length continued to increase roughly linearly over this range of reads (Figure 5 grey line). These results suggest that additional sequencing of this sample is unlikely to identify substantially more genes, but may continue to lengthen the existing sequences. Although in the absence of a sequenced genome it is not possible to accurately estimate how many genes are in fact present in the *O. fasciatus *transcriptome, we note that while several developmental genes of interest were identified in this study, others were not. (Tables 3, 4 and see below). Because these data suggest that we have sequenced these specific cDNA samples quite deeply, some form of specific target enrichment may be necessary for future attempts to discover additional genes not identified in this dataset.

**Table 3:** Selected signaling pathway genes identified in the *O. fasciatus *transcriptome.

				**Present in**:	
Pathway	# Hits	Hit ID (I/C/S)	Length (range)	Normalized	Non-Normalized
HEDGEHOG					
*cubitus interruptus*	3	I,S	225-906	Y	Y
*fused*	2	I	516-1582	Y	Y
*patched*	2	C, S	225-418	N	Y
*smoothened*	2	I	1270-1604	Y	Y
					
JAK/STAT					
*domeless*	1	I	4028	Y	Y
*hopscotch (janus kinase)*	3	I, C	473-2644	Y	Y
*Signal transducer and activator of transcription*	4	I	444-3270	Y	Y
					
NFKB/TOLL					
*cactus*	7	I, C	629-1748	Y	Y
*dorsal (Nuclear factor NF-kappa-B)*	2	I	1308-3926	Y	Y
*relish*	1	I	2650	Y	Y
*Toll*	11	I, C, S	215-4323	Y	Y
					
NOTCH					
*fringe*	1	I	877	Y	Y
*Hairless*	1	I	1053	Y	Y
*hairy (Enhancer of split/HES-1)*	1	I	2530	Y	Y
*mind bomb*	7 (6^†^)	I,C,S	335-1185	Y	Y
*Notch*	1	S	235	Y*	N
*Notchless*	1	I	2035	Y	Y
*Presenilin*	1	I	1661	Y	Y
*Serrate/Jagged*	2	S	246-300	Y*	Y
*strawberry notch*	7	I,S	191-3519	Y	Y
*Suppressor of Hairless*	3	I,C	375-697	Y	Y
					
WNT					
*armadillo*	5	I,S	348-3001	Y	Y
*dishevelled*	2	I	954-1321	Y	Y
*frizzled*	3	C,S	194-500	N	Y
*Wnt family (wingless, WNTs)*	6	C,S	207-508	Y	Y
					
TGF-BETA					
*decapentaplegic (BMP2/4)*	1	C	547	Y	Y
*glass bottom boat (BMP5/7)*	2	I	510-737	Y	Y
*SMADs (Mad, Smad2/3, Smad4/Medea)*	7	I,C	276-2276	Y	Y
*Type I Receptor (saxophone/thickveins/activin receptor type I)*	5	I,C	236-2466	Y	Y
*Type II Receptor (punt, wishful thinking)*	3	I	259-5038	Y	Y
					
RECEPTOR TYROSINE KINASES					
*Epidermal growth factor receptor*	7 (5^†^)	I,C,S	229-715	N	Y
*rhomboid*	2	C	229-602	N	Y
					
HORMONE SIGNALING (ECDYSONE, NUCLEAR HORMONE)					
*disembodied (ecdysteroidogenic P450)*	1	I	1835	Y	Y
*Ecdysone receptor*	2	I,C	231-1393	Y	Y
*E75*	3	I,S	257-649	Y	Y
*Ecdysone-induced protein 63E*	1	I	1479	Y	Y
*ecdysoneless*	1	I	4158	Y	Y
*Nuclear hormone receptor E78*	1	I	3150	Y	Y
*Nuclear hormone receptor HR3*	2	I	529-737	Y	Y
*phantom (cytochrome P450 306a1)*	2	C	344-575	N	Y
*shade (cytochrome 450 314A1)*	1	I	2125	Y	Y
*shadow (cytochrome 450 315A1)*	1	I	1650	Y	Y
*ultraspiracle nuclear receptor*	1	C	245	Y*	N
*without children*	2	I	1155-1357	Y	Y

Hit ID indicates if gene hits were found among isotigs (I), Cap3-assembled contigs (C), or unassembled singletons (S). Sequence length (range) indicates the shortest and longest S, C or I hit sequences for each gene. These results were generated by BLASTing the raw reads from the N and NN samples against the full assembly. When multiple sequences were obtained via name search, they were tested to see whether they could be made to form a contig with Sequencher or CLC Combined Workbench (see Methods). Asterisk indicates hits only present in normalized GS-FLX reads. X(Y^†^) indicates that the X sequences with hits could be assembled into Y contigs.

**Table 4:** Selected developmental process genes identified in the *O. fasciatus *transcriptome.

Process	# Hits	Hit ID (I/C/S)	Length (range)	Normalized	Non-Normalized
GERM PLASM					
*Argonaute 3*	2 (1^†^)	I	2042-2231	Y	Y
*germ cell-less*	2 (1^†^)	I	630-1817	Y	Y
*maelstrom*	1	I	994	Y	Y
*nanos*	1	I	1961	Y	Y
*piwi/aubergine*	1	I	2888	Y	Y
*pumilio*	2	I	424-2574	Y	Y
*staufen*	3	I	599-2100	Y	Y
*Tudor*	2	I	2719-3299	Y	Y
*vasa*	1	C	330	Y	Y
					
ANTERIOR-POSTERIOR DETERMINATION					

GAP					
*hunchback*	1	I	1429	Y	Y
*Kruppel*	1	S	250	N	Y
*ocelliless (orthodenticle)*	1	S	207	Y	N
					
TERMINAL GROUP					

*huckebein*	1	I	589	Y	Y
*torso-like*	2 (1^†^)	I,C	430-1868	Y	Y
					
PAIR RULE					

*fushi tarazu*	1	I	788	Y	Y
***hairy (Enhancer of split/HES-1)***	1	I	2530	Y	Y
*odd skipped*	1	C	346	N	Y
					
SEGMENT POLARITY					

***armadillo***	5	I,S	348-3001	Y	Y
***cubitus interruptus***	3	I,S	225-906	Y	Y
*engrailed*	1	S	227	Y*	N
***fused***	2	I	516-1582	Y	Y
*pangolin*	2	I,C	492-544	N	Y
***patched***	2	C, S	225-418	N	Y
***Wnt family (wingless, Wnts)***	6	C,S	207-508	Y	Y
					
DORSO-VENTRAL AXIS					
***cactus***	7	I, C	629-1748	Y	Y
***decapentaplegic (BMP2/4)***	1	C	547	Y	Y
*gastrulation-defective*	1	I	1773	Y	Y
*nudel*	4	I,S	322-1458	Y	Y
*pipe*	1	C	266	N	Y
*short gastrulation*	2	C	254-615	Y	Y
*snake*	1	I	1789	Y	Y
*spätzle*	2	I	993-3170	Y	Y
***Toll***	11	I, C, S	215-4323	Y	Y
					
MOLTING/METAMORPHOSIS					
*cuticular proteins (including CP 49Ae and adult cuticle protein)*	4	I,C	404-566	Y	Y
***disembodied (ecdysteroidogenic P450)***	1	I	1835	Y	Y
***Ecdysone receptor***	2	I,C	231-1393	Y	Y
***E75***	3	I,S	257-649	Y	Y
***Ecdysone-induced protein 63E***	1	I	1479	Y	Y
***ecdysoneless***	1	I	4158	Y	Y
*ftz transcription factor 1*	1	I	807	Y	Y
*hormone receptor 4*	2	I	1003-2114	Y	Y
*juvenile hormone acid methyltransferase*	5	I	548-2871	Y	Y
*juvenile hormone binding protein*	1	I	1099	Y	Y
*juvenile hormone epoxide hydrolase*	5	I,S	255-2859	Y	Y
*juvenile hormone esterase*	4	I	850-2382	Y	Y
*juvenile hormone esterase binding protein*	1	I	1057	Y	Y
*Juvenile hormone-inducible protein*	7	I	456-2757	Y	Y
*Methoprene-tolerant*	1	I	3415	Y	Y
***Nuclear hormone receptor E78***	1	I	3150	Y	Y
***Nuclear hormone receptor HR3***	2	I	529-737	Y	Y
***phantom (cytochrome P450 306a1)***	2	C	344-575	N	Y
***shade (cytochrome 450 314A1)***	1	I	2125	Y	Y
***shadow (cytochrome 450 315A1)***	1	I	1650	Y	Y
*takeout*	3	I	591-1011	Y	Y
***ultraspiracle nuclear receptor***	1	C	245	Y*	N
***without children***	2	I	1155-1357	Y	Y

Hit ID indicates if gene hits were found among isotigs (I), CAP3-assembled contigs (C), or unassembled singletons (S). Sequence length (range) indicates the shortest and longest S, C or I hit sequences for each gene. These results were generated by BLASTing the raw reads from the N and NN samples against the full assembly. When multiple sequences were obtained via name search, they were tested to see whether they could be made to form a contig with Sequencher or CLC Combined Workbench (see Methods). Asterisk indicates hits only present in normalized GS-FLX reads. X(Y^†^) indicates that the X sequences with hits could be assembled into Y contigs. **Boldface **indicates genes also present in Table 3.

**Figure 6:**
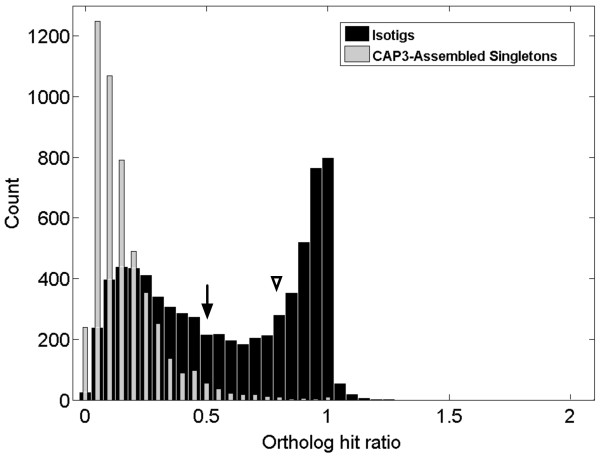
**Ortholog hit ratio analysis of isotigs and CAP3-reassembled singletons**. An ortholog hit ratio of one implies that a transcript has been assembled to its true full length. For isotigs (black), a majority (54.8%) appear to contain at least 50% of the full length transcript sequence (arrow), while over one-third (37.2%) appear to represent at least 80% of the full length transcript sequence (arrowhead). Most singletons (grey) represent much smaller percentages of full-length transcripts.

To address the second question, we employed a method proposed by O'Neil and colleagues [20] for addressing the question of how closely our sequences approached full-length transcripts. Their metric, the "ortholog hit ratio," compares the length of the newly discovered sequence that obtains a BLAST hit versus the full length of its top hit [20]. Thus, an ortholog hit ratio of one implies that a transcript has been assembled to its true full length, while values over one suggest insertions in the query sequence relative to its top BLAST hit. We note the caveat that many genes contain relatively poorly conserved regions that may fail to obtain a BLAST hit at all, causing the ortholog hit ratio to be an underestimate in these cases (Additional file 5). In our dataset, many of the *O. fasciatus *isotigs appear to be nearly fully assembled, while the singletons predictably tend to represent small portions of their top BLAST hit in RefSeq (Figure 6). In total, of the 7,219 isotigs with BLAST hits, 3,953 (54.8%) had ratios > 0.5 and 2,689 (37.2%) had ratios > 0.8.

**Figure 7:**
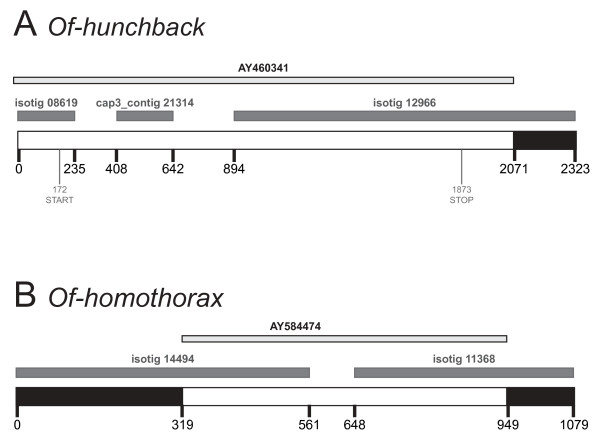
**The *O. fasciatus *transcriptome adds sequence data to existing GenBank accessions, which in turn improves annotation of transcriptome sequences**. *(A) *Extended contig for *Of-hunchback *(bottom), comprising the complete mRNA GenBank accession (top, light grey), two isotigs and one CAP3 contig from the transcriptome (middle, dark grey). The largest isotig provides an additional 252 bp of 3' UTR sequence to the GenBank sequence (black). Comparison with the GenBank sequence enabled isotig 08619 and cap3_contig 21314 to be assembled into the same contig. *(B) *Extended contig for *Of-homothorax *(bottom), with a partial mRNA GenBank accession (top, light grey) and two transcriptome isotigs (middle, dark grey). Both isotigs extend beyond the known GenBank sequence at the 3' and 5' ends, extending the known region by 449 bp in total (black). Both isotigs had been identified as *homothorax*, and because they did not overlap, they were classified as belonging to the same transcript rather than being paralogs. The GenBank sequence bridges an 87 bp gap between the isotigs, confirming that both sequences are fragments of a single gene.

We also asked, for those *O. fasciatus *sequences of developmental genes already present in GenBank that overlapped with transcriptome hits (n = 23), whether our transcriptome data provided any net gain in transcript sequence compared to the GenBank accession sequence. In 15/23 cases (68%), the transcriptome data extended the known sequence beyond that reported in GenBank by an average of 349 bp (range: 82-1,366 bp). In most cases, additional 3' sequence was obtained (Figure 7).

### Assessing the value of cDNA normalization

Reducing the representation of highly abundant transcripts (i.e. normalizing the cDNA) is often considered essential to capture sequence from genes expressed at lower levels, including many important developmental genes [see for example [55-57]]. However, we hypothesized that current next-generation sequencing technologies could provide sufficiently deep sequence to render normalization largely unnecessary for construction of *de novo *transcriptomes for comparative developmental biologists. To address this question, we assessed the relative contribution of the N and NN cDNA to our final assembly using several strategies.

**Figure 8:**
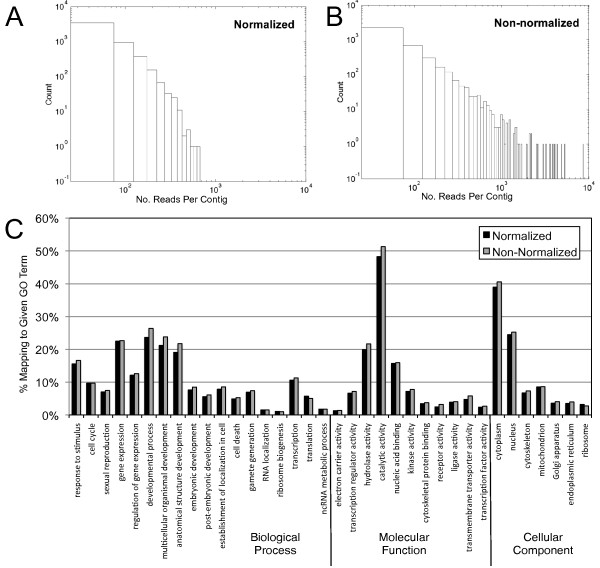
**Normalization decreases coverage of highly abundant genes, but does not change the GO term distribution of contigs**. In both samples, most contigs are composed of <10^2 ^reads. However, the non-normalized sample (*A*) contains contigs with many more reads per contigs than the normalized sample (*B*). In other words, normalization preferentially decreases the number of reads of those contigs with the most reads. *(C) *GO term distributions do not differ dramatically between pyrosequenced libraries of N versus NN cDNA. However, see Additional file 6 for exceptions. Column heights reflect the percentage of annotated sequences in each assembly that mapped to a given GO term. Note that the GO terms shown represent the results of mapping the N and NN reads against the complete assembly, rather than those obtained via independent assemblies of N and NN reads.

First, to test whether our normalization protocol successfully reduced the presence of highly abundant transcripts, we created separate assemblies from the N and NN cDNA samples (equalizing the total number of bases to reduce the contribution of additional sequence found in the NN sample). The N assembly contained a greater number of isotigs that were shorter on average than those in the NN assembly (Figure 2B). Additionally, more singletons were generated in the N assembly relative to the NN assembly (Table 2). Further, similar to the results obtained by Bellin and colleagues [27], we observed the predicted decrease in the maximum number of reads per contig in the N assembly compared to the NN assembly (Figure 8A, B), demonstrating that the normalization procedure successfully reduced the sequencing of highly abundant transcripts. These statistics, which could be interpreted to suggest that the N reads generated an inferior assembly, may result from the shorter average length of reads in the N sample (Figure 2A). Indeed, Newbler rejected 7.9% (30,780) of the N reads as too short, compared to only 1% (3,935) of the NN reads. However, these assembly statistics could also indicate greater heterogeneity in the N sample, which would suggest that normalization might increase the number of new genes identified.

To discriminate between these possibilities, we explored the contribution of the N and NN reads to the genes discovered in our full assembly. We used BLASTN to map one plate's worth of raw reads from the N sample and from the NN sample (equalized to contain the same number of base pairs) against the complete assembled transcriptome, with an e-value cut-off of 1e-4. We then explored the GO annotation of those genes hit exclusively by only one of these two samples. We observed similar overall GO term distributions between the N and NN samples (Figure 8C). We found that a small number of GO terms (n = 20) were significantly differentially represented in the two samples, albeit generally with very few sequences in each GO term (Additional file 6). For example, we were surprised to see that three of the four terms statistically over-represented in the N sample were related to ribosome function (14/750 (1.9%) of the N hits were annotated with 'ribosomal subunit', compared to 1/1124 (0.09%) NN hits; FDR-corrected *p*-value = 0.006). In contrast, several terms related to active transmembrane transport were over-represented in the NN sample (Additional file 6) possibly indicating that normalization may have reduced the representation of genes involved in certain basic metabolic processes.

As an additional way to investigate the contribution of the N and NN samples to identifying specific genes of interest for our studies, we manually examined the results of mapping the N and NN samples to the fully assembled transcriptome. Of the 79 genes of interest that we investigated, four (5.1%) were uniquely present in the N sample, whereas nine (11.4%) were uniquely present in the NN sample, and the remaining 66 (83.6%) were present in reads of both the N and NN samples (Tables 3, 4). Although this may be an artifact of sequencing depth (i.e. low-abundance genes of interest may be present in only one of the two cDNA samples simply due to sampling effects rather than the normalization protocol *per se*), our data suggest that the normalized cDNA sample did not contribute disproportionately to gene discovery.

### Gene discovery for developmental studies

The ultimate goal of this sequencing project was to identify a wide diversity of candidate genes involved in developmental processes. Traditionally, such gene discovery in "non-model" organisms has required degenerate PCR, which is labor-intensive, expensive, and prone to failure. The annotated transcriptome assembly we present here allows researchers to identify genes of interest via simple text searches, or via BLAST searches. To demonstrate the usefulness of these data for large-scale gene discovery, we report here the identification of several components from each of the seven widely studied metazoan signaling pathways (Table 3) as well as many genes involved in specific developmental processes (Table 4). We note that the majority of these gene fragments are of suitable length for immediate application of such widely used techniques as *in situ *hybridization and RNAi-based functional knockdown. In cases of functional experiments where full-length proteins are desirable, such as protein overexpression, RACE PCR will likely be required. Importantly, we note that many genes of interest were present among the singletons, many of which are long enough for immediate use as sequences for *in situ *hybridization probes or RNAi templates, emphasizing the importance of including these in NGS gene discovery studies.

Although we identified a diverse array of genes, some well-studied genes known to be expressed during embryogenesis were not easily identified in this study. For example, our BLAST results only contained three genes from the Hox cluster (*fushi tarazu, Antennapedia*, and *Abdominal-B*), although orthologs of all the canonical arthropod Hox genes are known to be present in *O. fasciatus *[58]. However, using the *O. fasciatus *Hox gene sequence fragments available from NCBI as a BLAST query against our transcriptome did reveal sequences for all Hox genes except *Sex combs reduced*. It is possible that these genes are expressed at very low levels during the developmental stages sampled here, suggesting that enrichment techniques may be necessary to more easily identify certain genes of interest. We do note, however, that *fushi tarazu*, the only Hox cluster gene not previously identified in *O. fasciatus*, was identified in both N and NN samples of this transcriptome dataset (Table 4).

### Case study: gene discovery for endocrine regulation of development

In addition to surveying the transcriptome for genes involved in embryonic patterning and other developmental processes, we asked whether we could also identify genes known to be employed in biological processes during postembryonic development of holometabolous insects. Recent studies have suggested that many of the genes used during holometabolous insect metamorphosis may also play important roles during embryogenesis in hemimetabolous insects [59,60]. To investigate this, we searched the *O. fasciatus *transcriptome for expression of key ecdysteroid- and juvenile hormone (JH)-related genes. We identified transcripts for many of the known ecdysteroid biosynthesis genes, including cytochrome P450 genes encoded by the *Drosophila *Halloween family, such as *shade *(CYP314A1), *shadow *(CYP315A1)*, phantom *(CYP306A1) and *disembodied *(CYP302A1) (Table 4). We also detected expression of ecdysone response genes. In particular, we identified many of the ecdysone-regulated genes that play key roles during molting and metamorphosis, including *E75*, *HR3*, and *HR4 *(Table 4). The presence of these genes in the ovaries and early embryos of *O. fasciatus *corroborates recent studies that implicate ecdysone-response genes in key developmental processes during embryogenesis [59-61]. As might be expected for a situation where ecdysone regulates embryonic development but not molting, transcripts encoding insect peptide hormones implicated in eclosion behavior, such as ecdysis-triggering hormone, eclosion hormone and crustacean cardioactive peptide, were not detected. JH biosynthesis and response genes were also isolated (Table 4). JH has been shown to play a role in promoting embryonic development and tissue maturation [62]. The expression of these genes, together with that of JH esterase and JH binding proteins, is consistent with previous studies implicating tight control of JH during embryogenesis [63].

## Conclusions

We have used 454 pyrosequencing to create an early developmental transcriptome for the milkweed bug *O. fasciatus *in the absence of a reference genome. Although genomic sequence data will be necessary in the future for linkage or *cis*-regulatory analyses, at the early stages of establishing new model organisms, one of the most important goals is often gene discovery. In this regard, while no transcriptome generated in this way can realistically be "complete" in the sense of containing full length transcripts for all expressed genes, we propose that for many evolutionary developmental biology studies, the approach described here is a useful one for fast, high-throughput gene discovery. A high priority for comparative developmental biology research is gene expression and function analyses. By sequencing at great depth and testing a variety of cDNA preparation methods (normalized, non-normalized, embryo- and ovary-specific), we have generated tens of thousands of gene sequences of sufficient lengths for the commonly used developmental techniques of *in situ *hybridization and RNAi-mediated gene knockdown. These data can also be used for phylogenetic, population genetic, and functional genomic applications, provide a starting point for identification of genomic regulatory sequences, and assist with assembly of hemipteran genomes sequenced in the future.

### Note added in Proof

While this article was in review, Kumar and Blaxter [64] published a comparison of *de novo *assemblers for 454 transcriptome data, and reported important shortcomings of Newbler v2.3 compared to other available assemblers. Specifically, the authors reported that Newbler v2.3 produced the smallest assembly (i.e. the smallest number of base pairs incorporated into contigs) of the assemblers tested. The authors argue that this poor performance is likely because Newbler v2.3 inexplicably discards portions of read overlap information. In contrast, a newer, currently unreleased version of Newbler, v2.5, produced the most complete assembly of all those tested. Kumar and Blaxter (2010) therefore strongly advise all *de novo *454 transcriptome assembly projects which have used Newbler v2.3 to recompute their assemblies with Newbler v2.5.

To address this concern, we obtained a pre-release version of Newbler v2.5 from Roche and reassembled the *O. fasciatus *data, again using the -nosplit flag. In contrast to Kumar and Blaxter (2010), we observed much less dramatic differences between the assemblies produced by Newbler v2.3 and Newbler v2.5 (Additional file 7). For example, Kumar and Blaxter (2010) report that Newbler v2.5 increased their total assembly size by 39% compared to Newbler v2.3. For the *O. fasciatus *data analyzed here, Newbler v2.5 increased the total assembly size by less than 1% (Additional file 7). Further, we observed very similar numbers of isogroups, isotigs, and singletons between the two assemblies (Additional file 7). We did observe a 16% increase in the number of contigs reported by Newbler v2.5, but this difference was markedly less than the 80% increase observed in the data analyzed by Kumar and Blaxter (2010). After BLASTing all of the assembled isotigs and cap3-assembled singletons against the RefSeq database, we identified a total of 10,886 unique BLAST hits, compared to 10,775 genes identified using Newbler v2.3.

These results suggest that, although we did observe a modest increase in assembly size using Newbler v2.5, the analyses presented in the current study are largely robust against differences between currently available versions of Newbler. One possible explanation for the difference between these results and those observed by Kumar and Blaxter (2010), is the greater sequencing depth performed in the current study. If in fact the poor performance of Newbler v2.3 involves discarding information in regions of low coverage, the fact that our dataset includes ~2.4x more reads than that analyzed by Kumar and Blaxter (2010) may explain the reduced improvement that Newbler v2.5 provided our dataset. We also suggest that the reduced number of genes identified via BLAST observed by Kumar and Blaxter (their Table five) may result from the fact that the authors excluded singletons from their analyses. If Newbler v2.3 indeed fails to assemble regions of low coverage and instead retains those reads as singletons, many genes of interest may only be present as singletons. Indeed, we observed many genes of interest exclusively represented as singletons (Tables 3 and 4). Thus, for the purpose of gene discovery, we emphasize that future *de novo *transcriptome projects should analyze singletons as an important source of useful gene sequence.

Although our results do not appear to be greatly sensitive to which version of Newbler is used, we agree with Kumar and Blaxter (2010) that future transcriptome project should use utilize the most current available version of Newbler, or whichever assembler algorithm they find most useful for their data.

## Methods

### Animal culture

The *O. fasciatus *specimens sequenced in this study were originally purchased from the Carolina Biological Supply Company (Burlington, NC) and were maintained in the laboratory on sunflower seeds under a 12h:12h light/dark cycle at 28°C.

### cDNA Synthesis

For our pilot study using the GS-FLX platform, total RNA was isolated from mature ovaries (Figure 1B) and from mixed-stage embryos representing the first three days of development (roughly 60% of embryogenesis at 28°C; Figure 1C, D) using TRIzol (Invitrogen), following the manufacturer's protocols. For each RNA sample, approximately 5 μg of cDNA was prepared using the SMART cDNA library construction kit (Clontech, CA, USA). The cDNA was normalized using Evrogen's Trimmer-Direct cDNA Normalization kit (Evrogen, Moscow, Russia), and subsequently digested with SfiI to partially remove the SMART adapters. The size distributions of total RNA and cDNA were assessed on 1.0% agarose gels following each step of the protocol.

To prepare cDNA for sequencing on the GS-FLX Titanium platform, we followed a modified version of the SMART cDNA protocol [65] that has been optimized for cDNA quality and yield from small quantities of total RNA. A helpful guide that formed the initial basis for the optimization of this protocol was once available online from Evrogen, but has since been removed. At the time these libraries were prepared, Roche had not yet provided a specific protocol for cDNA library preparation for 454 pyrosequencing. Subsequently, the company has released a cDNA protocol that requires approximately 500 ng of purified mRNA (typically requiring isolation of 10 to 50 μg of total RNA). While useful for larger tissue samples, the Roche cDNA preparation protocol is difficult to apply to samples in which RNA quantity is limiting, as is the case with many non-model organisms. The protocol we present here does not require the loss-prone step of mRNA purification, and we have found that it produces sufficient quantities of high-quality cDNA when 5 μl of the RNA (18S and 28S bands) can be visualized on a 1% agarose gel stained with ethidium bromide. Compared with the original SMART protocol, we have optimized the primers, PCR conditions, and downstream purification steps to maximize the yield of double-stranded cDNA required for 454 pyrosequencing. We initially optimized this protocol for Roche's original 454 library preparation protocol (not specific to cDNA), which required input of double-stranded DNA amounts of 2.5-10 μg (in our experience, typically 10-20 μg prepared cDNA as measured by UV absorbance). However, newer protocols from Roche require only 500 ng double-stranded cDNA, limiting the need for a secondary amplification step, as described here, for samples with highly limiting quantities of total RNA.

After separately isolating total RNA from mature ovaries (Figure 1B) and from each of the first three days of embryogenesis (Figure 1C, D) as described above, each RNA sample was treated with DNAse to remove potential genomic contamination. Equal amounts of each sample were then pooled for use as a template for first strand cDNA synthesis. Due to concerns that the poly(T) primer used in the SMART kit could interfere with pyrosequencing, the 3'-primer used was modified in two ways: (1) the poly(T) was interrupted every fourth base by the inclusion of a cytosine [sensu 30]; and (2) the primer contained an *Mme*I site which allowed most of the poly(T) to be removed during digestion. This 3'-primer (PD243Mme-30TC, 5'-ATT CTA GAG CGC ACC TTG GCC TCC GAC TTT TCT TTT CTT TTT TTT TCT TTT TTT TTT VN-3') was used during first strand synthesis and for all subsequent amplification steps. Because *Mme*I also cleaves relatively commonly within eukaryotic genes, it may not always be desirable to use this enzyme for library preparation. As an alternative, we have additionally found that a similar 3' primer containing an *Sfi*I cleavage site (PD243-30TC, 5'-ATT CTA GAG GCC ACC TTG GCC GAC ATG TTT TCT TTT CTT TTT TTT TCT TTT TTT TTT VN-3') is also effective in producing cDNA that yields high-quality 454 data (data not shown).

For first-strand synthesis, 3 μg of total RNA (in 6 μl) and 2 μl 3' primer (12 μM) were mixed and denatured at 65°C for 5 minutes, then placed on ice. Reverse transcription reactions using SuperScript II (Invitrogen) in the manufacturer's recommended buffer were performed for 50 minutes at 42°C using twice the recommended concentration of enzyme, 1 μl of Protector RNAse inhibitor (Roche) to avoid RNA degradation, 2 μl 5' primer (12 μM), 2 μl 10 mM DTT, and 1 μl 10 mM dNTPs. Template-switching essential for the SMART technique was achieved using a 5' primer (PD242, 5'-AAG CAG TGG TAT CAA CGC AGA GTG GCC ACG AAG GCC rGrGrG-3') with three RNA nucleotides at its 3' end, which contains an *Sfi*I site. Reactions were then heat-inactivated for 15 minutes at 70°C and diluted 1:5 in milliQ water in preparation for PCR amplification. Contrary to some expectations, SuperScript III reverse transcriptase (Invitrogen) may be substituted in this protocol with equivalent results (data not shown).

To maximize yield during cDNA amplification, the first round of amplification was conducted using a 2:2:1 mix (v:v:v) of Hemo KlenTaq (New England Biolabs), Phusion (New England Biolabs), and PfuTurbo (Stratagene) polymerases. This mixture of enzymes was determined empirically to provide the highest yield of cDNA with a range of input first-strand concentrations. Cesium KlenTaq AC (DNA Polymerase Technologies) and the hot start versions of Phusion and PfuTurbo polymerases in the same ratio may be also substituted at this step without sacrificing yield; this may produce fewer PCR artifacts in the final cDNA preparation. Buffer conditions (MgCl_2 _and DMSO) were also empirically optimized to maximize yield and minimize PCR artifacts. Reactions were performed in 100 μL total volume in 1X Phusion HF buffer, 1.5 μL polymerase mix, 5 μL first-strand cDNA (previously diluted 1:5 in H_2_O), 1 μL 3' primer (PD243Mme-30TC, 12 μM), 1 μL 5' primer (PCRIIA, 5'-AAG CAG TGG TAT CAA CGC AGA GT-3', 12 μM), and a final concentration of 1% DMSO, 1.5 mM MgCl_2 _(in addition to the MgCl_2 _already present in the HF buffer), and 200 μM dNTPs. Reactions were cycled with the following program: 1 minute at 95°C, followed by 16-20 cycles of 30 seconds at 95°C (see below for determining optimal number of cycles), 30 seconds at 66°C, and 3 minutes at 72°C, and a final 10 minutes at 72°C. After cooling to room temperature, 10 μL 3M NaOAc pH 5.5 was added to each 100 μL secondary PCR reaction followed by purification with the QiaQuick PCR purification kit (Qiagen) using the manufacturer's recommended protocol. For all purification steps, samples were eluted with TM buffer (10 mM Tris-HCl pH 8.5, 1 mM MgCl_2_) to prevent strand separation of double-stranded cDNA.

To produce sufficient cDNA for sequencing, Advantage 2 (Clontech) polymerase was used under the manufacturer's recommended conditions during the second round of amplification using the same primer concentrations and 1 μl of undiluted primary PCR product. We recommend testing a range of dilutions of the primary PCR product to obtain the desired quantity of amplified cDNA in 9-10 PCR cycles. In cases of highly limiting RNA concentration, we have also found that a secondary PCR reaction using a 1:1:1 mix of Phusion, Cesium KlenTaq AC, and Deep Vent (exo-) (New England Biolabs) polymerase in ThermoPol reaction buffer supplemented with 1.5 mM MgSO_4 _and 1% DMSO produces the highest yield of secondary PCR product (note that this polymerase mix does not produce optimal results when used for first-round amplification). Secondary PCR reactions were cycled using the same parameters as the primary PCR but running for approximately 10 cycles.

To prevent overcycling during both rounds of PCR amplification, each reaction was prepared in duplicate, and one reaction was spiked with 1 μl of 1:750 SybrGreen I (Invitrogen). The spiked reactions were monitored in real time on an Mx3005P QPCR machine (Stratagene Inc.), and the samples were removed when amplification began to plateau. To increase the representation of double-stranded cDNA, two cycles of "chase PCR" were conducted following each round of cDNA amplification after the optimal number of cycles had been reached. Excess primers were added (1.5 μL of each, 12 μM primer per 100 μL reaction), and each reaction was subjected to two additional non-denaturing cycles of 1 minute at 77°C, 1 minute at 65°C, and 3 minutes at 72°C, followed by a 10 minute extension at 72°C.

Following the second round of amplification and PCR purification, the cDNA samples were double-digested with *Sfi*I and *Mme*I (40 and 26 units per 150 μl reaction, respectively). cDNA species <500 bp were then removed using Chroma Spin 400 columns (Clontech) which had been equilibrated with TM buffer following the manufacturer's protocol. It should be noted that the Chroma Spin column protocol suggested in the Clontech SMART cDNA kit is non-optimal, and that following the protocol provided with the separately purchased columns is less labor-intensive and produces a higher yield of size-selected cDNA. Equilibration of Chroma Spin columns is critical for maximizing the yield of double-stranded cDNA as required by the Roche library preparation protocols. Following size selection, cDNA was blunt-ended with the NEB Quick Blunting kit (New England Biolabs) and purified once more with the QiaQuick kit. After each step of cDNA synthesis, the size distribution was checked on 1.0% agarose gels, and the cDNA samples were quantified using a Qubit (Invitrogen), after observing that the NanoDrop 1000 (Thermo Scientific) did not reliably quantify ds-cDNA (C. Dunn, personal communication).

To prepare normalized cDNA for GS-FLX Titanium sequencing, 1 μl of the twice-amplified, purified cDNA sample described above was subjected to Evrogen's DSN-treatment protocol, followed by a single round of further amplification, *Sfi*I/*Mme*I digestion, and size selection. Approximately 5 μl of normalized and non-normalized cDNA were synthesized.

### 454 Titanium Pyrosequencing

For the pilot study using the GS-FLX platform, EnGenCore (University of South Carolina) conducted the final steps of library preparation, including nebulization, adaptor-ligation, and sequencing of each sample (¼ plate each). For sequencing using the Titanium platform, the samples were nebulized, adaptor-ligated, and pyrosequenced by the Institute for Genome Science and Policy DNA Sequencing Facility (Duke University).

### Sequence Assembly

Raw reads were assembled using the cDNA assembly algorithm of Newbler v2.3 (Roche) with default assembly parameters. An adaptor-trimming step was included in the assembly (the "-v" flag), and the "-nosplit" flag was also used to reduce the generation of extremely short contigs that might otherwise have been created. All of the raw reads generated in this study have been submitted to the NCBI Short Read Archive (Study Accession Number: SRP002610.1).

Because redundancy was observed among the singletons generated by Newbler v2.3, the singletons were reassembled using CAP3 [48], with '-z' option set to 1. Prior to this secondary assembly, the singletons were screened for adaptor sequences using both cross_match [66-68] and a custom python script (Casey Dunn, personal communication), We note that Newbler can also be used to produce a .fasta and corresponding .qual files of trimmed reads using the '-tr' option. The final assembly thus consists of three types of sequences: Newbler-assembled sequences, cap3_contigs, and cap3_singlets, all of which were subjected to subsequent analyses.

### Sequence Annotation

Sequences were first mapped against the RefSeq Protein database [[69], downloaded from ftp://ftp.ncbi.nih.gov/blast/db/ on April 27, 2010] using BLASTX. All BLAST searches were conducting using BLAST v2.2.23+ [70] with an e-value cut-off of 1e-10. We then used Blast2GO v1.2.7 [54] to retrieve the Gene Ontology (GO) [71] terms and their parents associated with the top 20 BLAST hits for each sequence. To avoid potentially double-counting sequences that might represent un-assembled portions of the same transcript, a custom python script ("transcriptome_blast_summarizer.py", available at http://www.extavourlab.com/protocols/index.html) was used to identify sequences with identical top BLAST hits prior to GO annotation. If multiple sequences hit non-overlapping portions of the same top BLAST hit, we used the conservative assumption that these sequences represented unassembled portions of the same transcript, and therefore only tallied the GO terms of one of these sequences. However, if multiple sequences hit overlapping portions of the same top BLAST hit, we considered these sequences potential paralogs and retained them all. Thus, the counts of sequences in each GO term only include one sequence per top BLAST hit, unless the multiple sequences mapped to overlapping portions of the same BLAST hit. These counts were used to compare the distribution of sequences among specific GO terms between the transcriptomes of *O. fasciatus *and the *Drosophila melanogaster *genome. For this comparison, we used a precomputed GO annotation of the *D. melanogaster *genome [72].

The FASTA formatted transcriptome data set file was examined in TextWrangler (v. 3.1, Bare Bones Software, Inc.). Candidate genes were sought via whole gene names and, where possible, via the gene name abbreviations, while avoiding irrelevant hits. The FASTA header annotation of transcriptome sequences includes the top 20 BLASTx hits to the RefSeq database as described above.

Sequencher (v4.8, Gene Codes Corporation; default settings: minimum 20 bp overlap between sequences, ≥85% sequence identity) and CLC Combined Workbench (v5.6.1, CLC Bio) were used to examine whether transcriptome sequences could be further assembled.

### Estimating sequencing depth

To estimate how thoroughly our sequencing efforts sampled the *O. fasciatus *transcriptome, eight progressively larger subsets of the reads were independently assembled. The total number of genes was then identified via BLASTX. For these smaller assemblies, reads from one plate each of normalized and non-normalized reads were combined in random order and sampled without replacement. For each assembly, we BLASTed the longest isotig of each isogroup, and all of the singletons, against the SwissProt database [[73], downloaded from ftp://ftp.ncbi.nih.gov/blast/db/ on April 21, 2010]. We used the relatively small SwissProt database in order to reduce computation time. However, the absolute values of BLAST hits against this database are likely to be underestimates of those values that would have been obtained from a larger database such as RefSeq or nr. If multiple isotigs or contigs hit non-overlapping portions of the same top BLAST hit, only one of these sequences was counted. However, because frequent cases of identical, unassembled singletons were observed, we counted only one singleton per top BLAST hit, regardless of whether these hits overlapped or not.

We used a custom python script to calculate the ortholog hit ratio. This script, "ortholog_hit_ratio_calculator.py" is available at http://www.extavourlab.com/protocols/index.html).

### Assessing the importance of cDNA normalization

To assess the relative contribution of cDNA normalization to the quality of our assembly, the screened, raw reads from both normalized (N) and non-normalized (NN) samples were mapped against the complete assembly of all reads using the BLASTN algorithm [70] with an e-value cut-off of 1e-4. Based on these results, the Fisher's Exact Test was used to identify over- and under-represented terms in each gene list. This test was performed using Blast2GO (two-tailed, removing double IDs so that only those genes hit uniquely by either N or NN reads were considered). The BLASTN results were also investigated using text searches to find whether certain genes of interest were present in only one of the two cDNA samples.

## Competing interests

The authors declare that they have no competing interests.

## Authors' contributions

BEC helped design the research, performed the experiments, collected and analyzed the data, and wrote the manuscript. NS contributed new protocols and helped write the manuscript. KAP helped analyze the data and write the manuscript. YS helped analyze the data and write the manuscript, and obtained funding for the research. SR helped design the research and review the manuscript, and obtained funding for the research. CE proposed the idea for the research, helped design the research and analyze the data, wrote the manuscript and obtained funding for the research. All authors read and approved the final manuscript.

## Supplementary Material

Additional file 1 **Normalized sample did not perform equally in pilot and full sequencing runs**. (*A*) For the normalized sample, the read lengths of the full plate sequencing runs (white) were shorter than those obtained by the 1/8 plate run (grey). (*B*) The read length distribution of the non-normalized sample was comparable for both 1/8 plate (grey) and full plate (white) sequencing runs. Click here for file

Additional file 2 **Distribution of average coverage (reads/bp) within contigs in the *O. fasciatus *transcriptome**. The coverage within contigs is calculated by dividing the total number of base pairs contained in the reads used to construct a contig by the length of that contig. Note that Newbler v2.3 discards those contigs <100 bp. Click here for file

Additional file 3 **RT-PCR validation of bioinformatically predicted multiple isoforms**. (*A*) Schematic of experimental design. Ten isogroups were randomly selected, each containing exactly two isotigs that differed by the presence/absence of a single contig. PCR primers were designed to flank the differing region. (*B*) Band sizes predicted by Newbler v2.3 for ten randomly selected isogroups containing exactly two isotigs. *(C) *Agarose gel following RT-PCR using primers against the sequences described in (*B*). Ladder sizes are given in base pairs on the left. Blue arrowheads: bands of the sizes predicted by Newbler v2.3; red arrowheads: bands not predicted by Newbler v2.3. Click here for file

Additional file 4 **Identity of taxa with top BLAST hits**. "Isotigs" refers only to the longest isotig of each isogroup; "Singletons" refers to the Newbler-generated singletons after secondary CAP3 assembly. The category "other" is the summation of all those species obtaining very low numbers of BLAST hits. Click here for file

Additional file 5 ***O. fasciatus *assembly isotigs have ortholog hit ratios similar to predictions from fully genome-sequenced databases**. When isotigs from the *O. fasciatus *transcriptome are BLASTed against the RefSeq protein database, ortholog hit ratios show a similar profile to those obtained when the complete *Acyrthosiphon pisum *gene prediction set (downloaded from http://www.aphidbase.com/aphidbase/downloads/) is BLASTed against the predicted gene set of *Drosophila melanogaster *(r5.28 downloaded from ftp://ftp.flybase.net/genomes/Drosophila_melanogaster/) with an e-value cut-off of 1e-10. Click here for file

Additional file 6 **GO terms enriched in Normalized (N) and Non-Normalized (NN) cDNA samples**. N (assembly generated from full plate of normalized cDNA) and NN (assembly generated from an equalized number of base pairs of non-normalized cDNA) reads were BLASTed against the full transcriptome assembly, and the results were used to generate "test" and "reference" sets for a Fisher's Exact Test. FDR: false discovery rate. Click here for file

Additional file 7 **Comparison of *de novo *transcriptome assemblies produced by Newbler v2.3 and Newbler v2.5**. Number of BLASTx hits reflects a search against RefSeq Protein database with an e-value cut-off value of 1e-10. Click here for file
