# Supplementary figures and images for: The maternal and early embryonic transcriptome of the milkweed bug Oncopeltus fasciatus
Source: BMC Genomics. 2011 Jan 25;12:61. doi: 10.1186/1471-2164-12-61 (PMC3040728; doi:10.1186/1471-2164-12-61)

**A**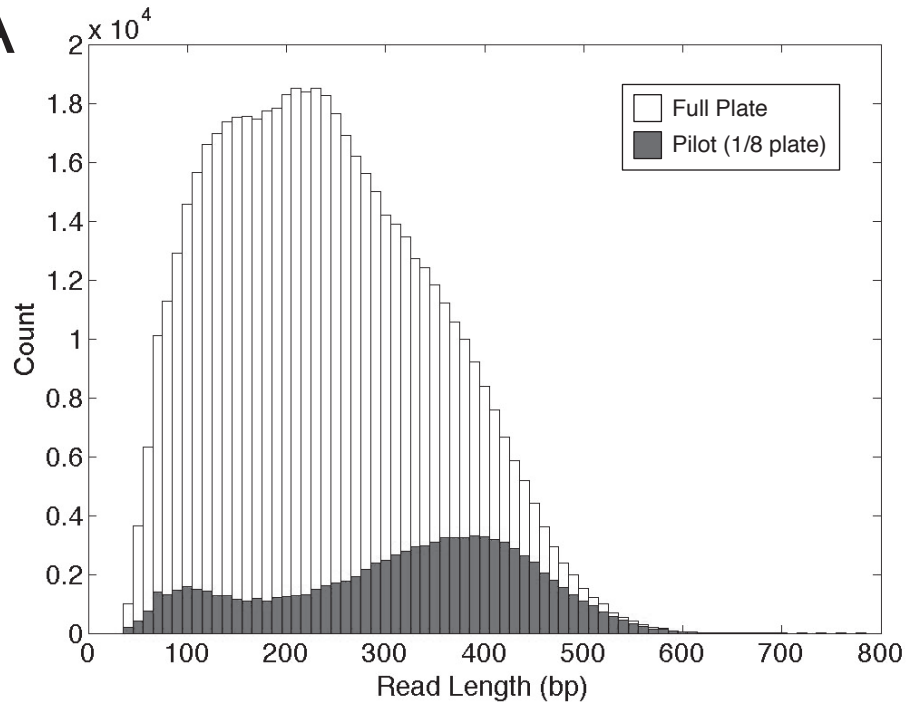**B**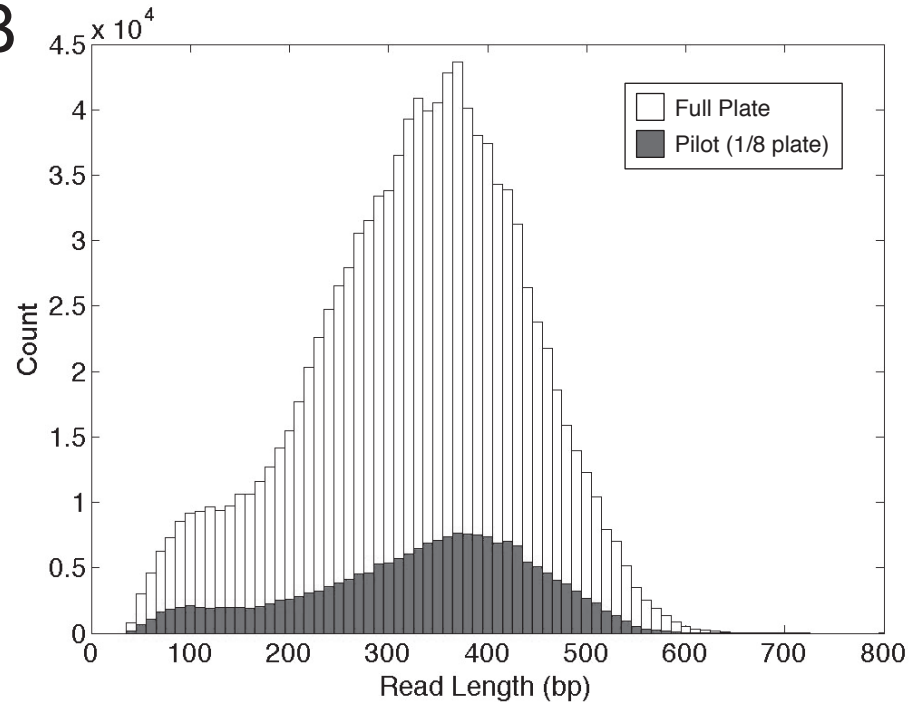

Supplement: Additional file 1 — Normalized sample did not perform equally in pilot and full sequencing runs. (A) For the normalized sample, the read lengths of the full plate sequencing runs (white) were shorter than those obtained by the 1/8 plate run (grey). (B) The read length distribution of the non-normalized sample was comparable for both 1/8 plate (grey) and full plate (white) sequencing runs. [file 1471-2164-12-61-S1.PDF]

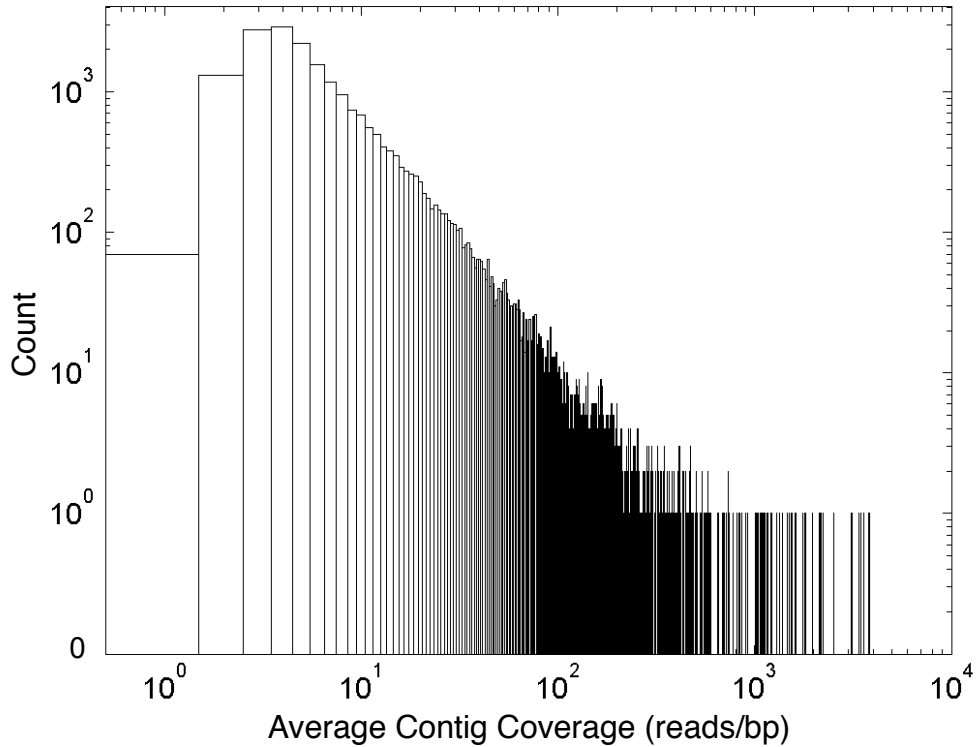

Supplement: Additional file 2 — Distribution of average coverage (reads/bp) within contigs in the O. fasciatus transcriptome. The coverage within contigs is calculated by dividing the total number of base pairs contained in the reads used to construct a contig by the length of that contig. Note that Newbler v2.3 discards those contigs <100 bp. [file 1471-2164-12-61-S2.PDF]

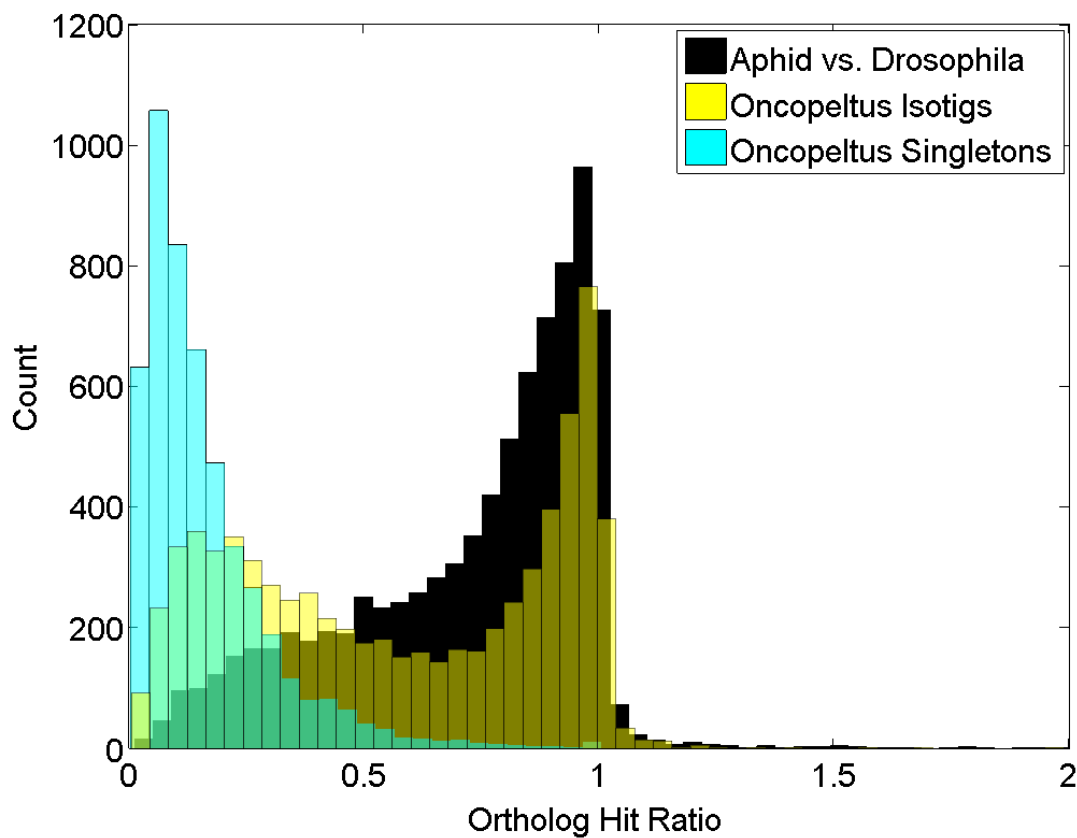

Supplement: Additional file 5 — O. fasciatus assembly isotigs have ortholog hit ratios similar to predictions from fully genome-sequenced databases. When isotigs from the O. fasciatus transcriptome are BLASTed against the RefSeq protein database, ortholog hit ratios show a similar profile to those obtained when the complete Acyrthosiphon pisum gene prediction set (downloaded from http://www.aphidbase.com/aphidbase/downloads/) is BLASTed against the predicted gene set of Drosophila melanogaster (r5.28 downloaded from ftp://ftp.flybase.net/genomes/Drosophila_melanogaster/) with an e-value cut-off of 1e-10. [file 1471-2164-12-61-S5.PDF]
