# Supplementary material for: The maternal and early embryonic transcriptome of the milkweed bug Oncopeltus fasciatus
Source: BMC Genomics. 2011 Jan 25;12:61. doi: 10.1186/1471-2164-12-61 (PMC3040728; doi:10.1186/1471-2164-12-61)

A

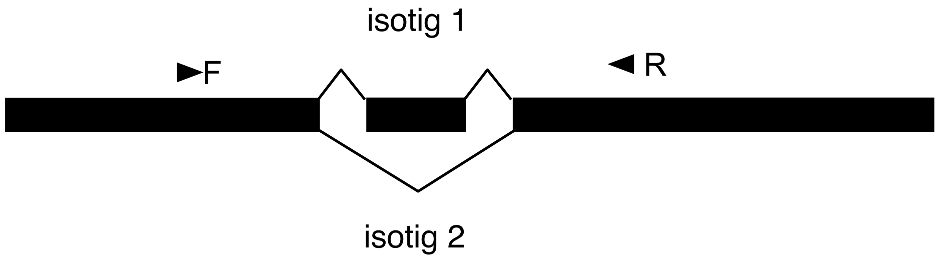

B

Lane	Isogroup	Predicted Band 1	Band 1 Present?	Predicted Band 2	Band 2 Present?	Additional Bands Present?
1	935	625	Y	319	Y	Y
2	948	657	Y	347	Y	Y
3	984	756	Y	628	Y	N
4	1045	759	Y	609	Y	N
5	1082	789	Y	623	Y	N
6	1133	701	N	579	Y	Y
7	1134	652	Y	519	N	N
8	1144	659	Y	515	Y	N
9	1162	759	Y	585	Y	Y
10	1179	700	Y	437	Y	Y

C

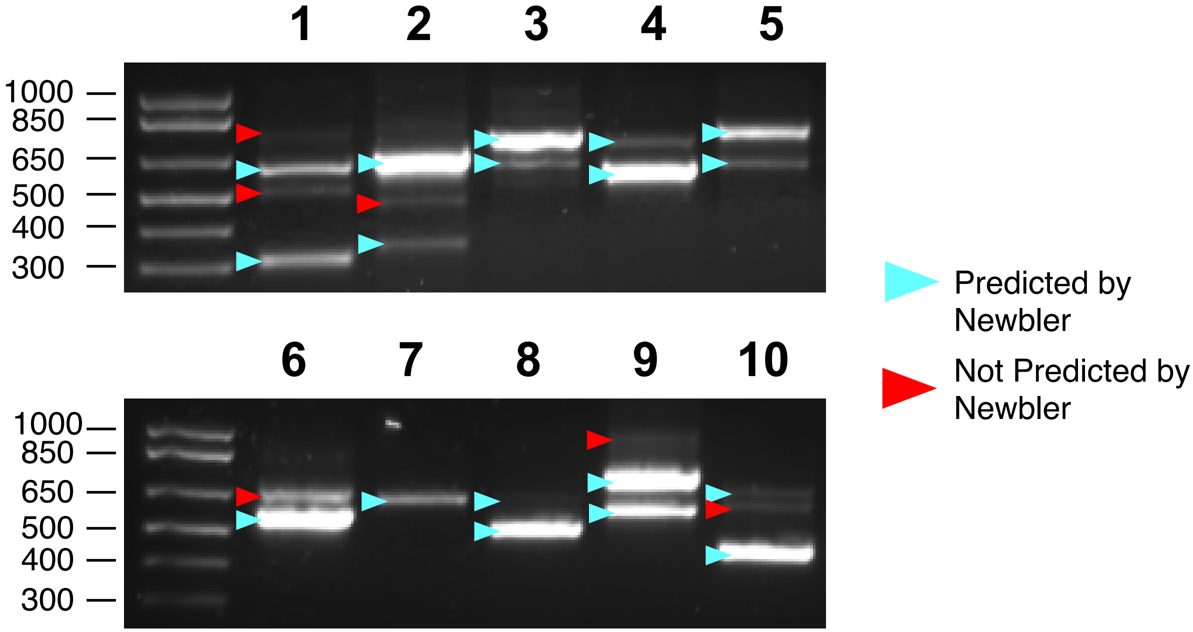

Supplement: Additional file 3 — RT-PCR validation of bioinformatically predicted multiple isoforms. (A) Schematic of experimental design. Ten isogroups were randomly selected, each containing exactly two isotigs that differed by the presence/absence of a single contig. PCR primers were designed to flank the differing region. (B) Band sizes predicted by Newbler v2.3 for ten randomly selected isogroups containing exactly two isotigs. (C) Agarose gel following RT-PCR using primers against the sequences described in (B). Ladder sizes are given in base pairs on the left. Blue arrowheads: bands of the sizes predicted by Newbler v2.3; red arrowheads: bands not predicted by Newbler v2.3. [file 1471-2164-12-61-S3.PDF]
