# Supplementary material for: The maternal and early embryonic transcriptome of the milkweed bug Oncopeltus fasciatus
Source: BMC Genomics. 2011 Jan 25;12:61. doi: 10.1186/1471-2164-12-61 (PMC3040728; doi:10.1186/1471-2164-12-61)
Supplement: Additional file 4 — Identity of taxa with top BLAST hits. "Isotigs" refers only to the longest isotig of each isogroup; "Singletons" refers to the Newbler-generated singletons after secondary CAP3 assembly. The category "other" is the summation of all those species obtaining very low numbers of BLAST hits. [file 1471-2164-12-61-S4.PDF]

Top BLAST hit taxa	Isotigs	Singletons (CAP3-assembled)	Total
Holometabola	4,311	2,650	6,961
Hemimetabola	2,156	1,640	3,796
Deuterostomes	358	234	592
Non-hexapod arthropods	62	58	120
Non-bilaterian metazoa	47	48	95
Non-metazoa	21	0	21
Others	264	331	595
