# Supplementary material for: The maternal and early embryonic transcriptome of the milkweed bug Oncopeltus fasciatus
Source: BMC Genomics. 2011 Jan 25;12:61. doi: 10.1186/1471-2164-12-61 (PMC3040728; doi:10.1186/1471-2164-12-61)
Supplement: Additional file 6 — GO terms enriched in Normalized (N) and Non-Normalized (NN) cDNA samples. N (assembly generated from full plate of normalized cDNA) and NN (assembly generated from an equalized number of base pairs of non-normalized cDNA) reads were BLASTed against the full transcriptome assembly, and the results were used to generate "test" and "reference" sets for a Fisher's Exact Test. FDR: false discovery rate. [file 1471-2164-12-61-S6.PDF]

GO Term	FDR	# Normalized (total n = 750)	# Non-Normalized (total n = 1124)	Enriched
establishment of localization (GO:0051234)	0.0253	107	227	NN
transport (GO:0006810)	0.0283	106	224	NN
transporter activity (GO:0005215)	0.0475	50	121	NN
ATPase activity (GO:0016887)	0.0174	35	99	NN
establishment of localization in cell (GO:0051649)	0.0411	32	88	NN
vesicle-mediated transport (GO: 0016192)	0.0111	16	61	NN
active transmembrane transporter activity (GO:0022804)	0.0001	11	62	NN
ATPase activity, coupled to movement of substances (GO:0043492)	0.0001	7	51	NN
hydrolase activity, acting on acid anhydrides, catalyzing transmembrane movement of substances (GO:0016820)	0.0001	6	52	NN
primary active transmembrane transporter activity (GO:0015399)	0.0001	6	49	NN
P-P-bond-hydrolysis-driven transmembrane transporter activity (GO:0015405)	0.0001	6	49	NN
ATPase activity, coupled to transmembrane movement of substances (GO:0042626)	0.0001	6	49	NN
GTPase activator activity (GO:0005096)	0.0480	6	31	NN
ATPase activity, coupled to transmembrane movement of ions (GO:0042625)	0.0475	2	20	NN
ATPase activity, coupled to transmembrane movement of ions, phosphorylative mechanism (GO:0015662)	0.0325	1	18	NN
lipid transporter activity (GO:0005319)	0.0475	0	13	NN
cytosolic part (GO:0044445)	0.0031	21	4	N
structural constituent of ribosome (GO:0003735)	0.0282	17	4	N
ribosomal subunit (GO:0033279)	0.0059	14	1	N
large ribosomal subunit (GO:0015934)	0.0381	9	0	N
