# Supplementary material for: The maternal and early embryonic transcriptome of the milkweed bug Oncopeltus fasciatus
Source: BMC Genomics. 2011 Jan 25;12:61. doi: 10.1186/1471-2164-12-61 (PMC3040728; doi:10.1186/1471-2164-12-61)
Supplement: Additional file 7 — Comparison of de novo transcriptome assemblies produced by Newbler v2.3 and Newbler v2.5. Number of BLASTx hits reflects a search against RefSeq Protein database with an e-value cut-off value of 1e-10. [file 1471-2164-12-61-S7.PDF]

	Newbler v.3	Newbler 2.5
Total bases assembled	19,921,298	20,096,403
Isogroups ("genes")	16,629	16,849
Isotigs ("transcripts")	21,097	20,985
Isotig N50	1,735	1,651
Mean # isotigs per isogroup	1.3	1.2
Contigs ("exons")	22,235	25,955
Mean # contigs per isotig	1.9	1.8
Singletons (singletons after secondary cap3 assembly)	178,770 (112,531)	168,807 (114,487)
Total # unique genes identified BLASTx	10,775	10,886
